# Analysis of the Mechanical Behavior, Creep Resistance and Uniaxial Fatigue Strength of Martensitic Steel X46Cr13

**DOI:** 10.3390/ma10040388

**Published:** 2017-04-06

**Authors:** Josip Brnic, Sanjin Krscanski, Domagoj Lanc, Marino Brcic, Goran Turkalj, Marko Canadija, Jitai Niu

**Affiliations:** 1 Department of Engineering Mechanics, Faculty of Engineering, University of Rijeka, Vukovarska 51, 51000 Rijeka, Croatia; sanjin.krscanski@riteh.hr (S.K.); dlanc@riteh.hr (D.L.); mbrcic@riteh.hr (M.B.); turkalj@riteh.hr (G.T.); markoc@riteh.hr (M.C.); 2 School of Materials Science and Engineering, Harbin Institute of Technology, XiDaZhi Street 92#, Nangang District, Harbin 150001, China; niujitai@163.com; 3 School of Materials Science and Engineering, Henan Polytechnic University, No 2001, Century Avenue, Jiaozuo 454003, China

**Keywords:** analysis, mechanical properties, short-time creep, fatigue, X46Cr13 steel, 81.05.Bx, 81.70.Bt, 81.40Np, 62.20.Hg, 62.20.me, 62.20.-x

## Abstract

The article deals with the analysis of the mechanical behavior at different temperatures, uniaxial creep and uniaxial fatigue of martensitic steel X46Cr13 (1.4034, AISI 420). For the purpose of considering the aforementioned mechanical behavior, as well as determining the appropriate resistance to creep and fatigue strength levels, numerous uniaxial tests were carried out. Tests related to mechanical properties performed at different temperatures are presented in the form of engineering stress-strain diagrams. Short-time creep tests performed at different temperatures and different stress levels are presented in the form of creep curves. Fatigue tests carried out at stress ratios R=0.25 and R=−1 are shown in the form of S–N (fatigue) diagrams. The finite fatigue regime for each of the mentioned stress ratios is modeled by an inclined log line, while the infinite fatigue regime is modeled by a horizontal line, which represents the fatigue limit of the material and previously was calculated by the modified staircase method. Finally, the fracture toughness has been calculated based on the Charpy V-notch impact energy.

## 1. Introduction

The properties of the materials used in structural design need to be correlated with the purpose for which the structure is designed [1]. A structural design procedure, the manufacture of the structure, as well as structure maintenance must be able to ensure smooth and safe operation without any failure. However, many failures can occur during structure service life, and they can be caused by factors, such as corrosion, inadequate loading, wear, failure presence in the material, the improper use of material, poor design, poor structural assembly, manufacturing defects, unforeseen operating conditions, inadequate maintenance, untimely and inadequate control, etc. [2,3]. The failure modes are usually listed as fatigue, creep, fracture, buckling, corrosion, elastic deformation, etc. [4]. This study primarily considers two of the mentioned failure modes; the former is the creep behavior at different temperatures and different stress levels, and the latter is uniaxial fatigue at prescribed stress ratios. The creep phenomenon is usually defined as a time-dependent behavior where strain continuously increases while the stress (load) is kept constant. It is usually appreciable at temperatures above 40% of the melting temperature of the material [5]. The following part of this paper is concerned with some of the recent research dealing with the steel X46Cr13. The work in [6] focuses on the lifetime reduction of cyclically-loaded X46Cr13 steel constantly exposed to highly corrosive CO_2_-saturated hot thermal water at 60 °C. Furthermore, the corrosion behavior of X46Cr13 martensitic stainless steel depends strongly on the applied heat treatment, which was considered in [7]. Since steel X46Cr13 is usually used in cutlery fabrication requiring corrosion resistance and high hardness, an analysis is presented in [8] with reference to the identification of the heat treatment parameters influencing the corrosion resistance of the martensitic stainless steel. Furthermore, the influence of the microstructure and surface treatment on the corrosion resistance of martensitic steel was studied, and the results are consequently presented in [9]. A brief research work of the influence of process parameters on the quality of thermally sprayed material coating is given in [10] since the final coating quality is strongly related to the spray parameters’ definition, such as oxygen pressure, fuel gas type, etc. Sliding wear properties and corrosion resistance related to X46Cr13 steel were tested, and this problem is treated in [11]. Mechanical data on this steel are very scarce in the literature. The motivation for this investigation and preparing of the paper is to provide an insight into the behavior of materials under different temperature conditions. These data can be of interest for designers of the structures since this steel alloy can be used for pump parts, roller bearings, etc.

## 2. Experimental Procedures

### 2.1. Tested Material

Experimental results elaborated in this paper are related to the chromium martensitic stainless steel X46Cr13. The as-received material, as stated in the certificate of the supplier, was annealed and is a 16-mm cold drawn round bar, whose chemical composition is shown in Table 1. However, the certificate of the material does not provide insight into the details that can explain how the delivered state of the material is achieved. This steel is commonly recognized as a high hardness material in conjunction with good corrosion resistance. Due to its high hardness, it is also well suited for the production of roller bearings, cutting tools, etc. Usually, it is most widely used in the quenched and tempered condition. As mentioned, this material may be used in mechanical, civil and industrial engineering, means for transport, etc. In particular, it can be used in the manufacturing of pump parts, machined metal parts, shaft, gears, tie rods, screws, etc. Pumps can be exposed to high corrosive conditions, such as corrosive thermal water present in geothermal power plants. The results of this research related to uniaxial fatigue testing provide an insight into the material fatigue behavior under normal environmental conditions at room temperature. However, this material can also be used for the production of valves and molds for the production of plastics, the cutting tools industry, etc.

### 2.2. Equipment, Specimens, Testing Procedures and Standards

**Figure 1:**
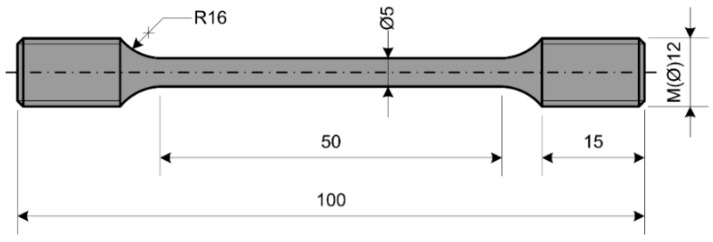
Specimen’s geometry (mm): tensile test.

This research includes several types of tests, testing procedures and standards. However, the standards define the geometry of the specimen used in the test, as well as the testing procedure. Uniaxial tests that included the determination of the material properties and the material creep behavior were performed using a 400-kN capacity material testing machine. Measurements were conducted with a macro extensometer and a high temperature extensometer. In fatigue testing, the servopulser of ±50-kN capacity was used, while in impact energy determination, a Charpy impact machine (150 J; 300 J) was used. The type of the specimen (shape and geometry) used in uniaxial testing in order to determine the stress-strain diagrams at room and elevated temperatures, as well as material creep behavior is shown in Figure 1.

**Figure 2:**
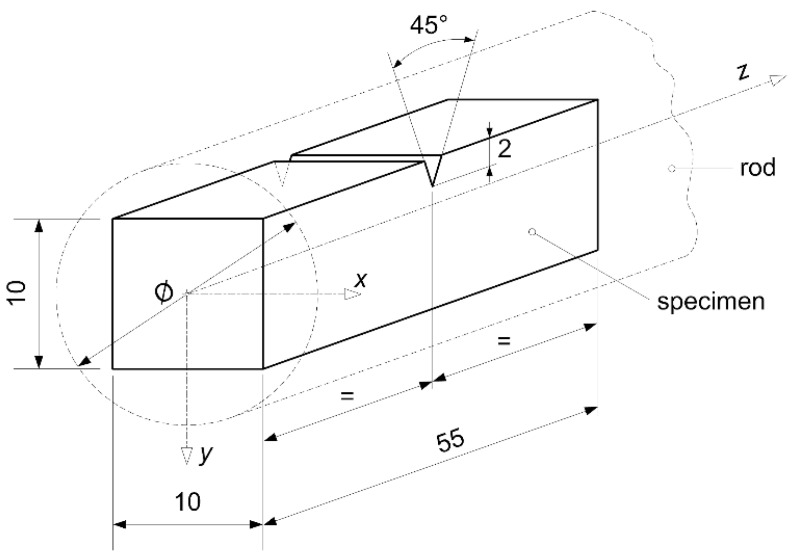
Charpy V-notch specimen (mm).

Specimens were machined from the 16-mm steel rod in accordance with ASTM: E 8M-15a. Tensile tests (uniaxial tensile testing/procedures) related to the determination of the stress-strain diagram at room temperature were carried out in accordance with the mentioned ASTM: E 8M-15a standard, while those related to high temperatures were performed in accordance with the ASTM: E 21-09 standard. The modulus of elasticity was determined in accordance with the ASTM: E 111 standard. Creep tests were carried out in accordance with the ASTM: E 139-11 standard. Subsequently, the geometry of the specimen used in Charpy impact energy determination is presented in Figure 2. Charpy tests were carried out on the Charpy V-notch specimens, machined in accordance with the ASTM: E 23-12c standard.

**Figure 3:**
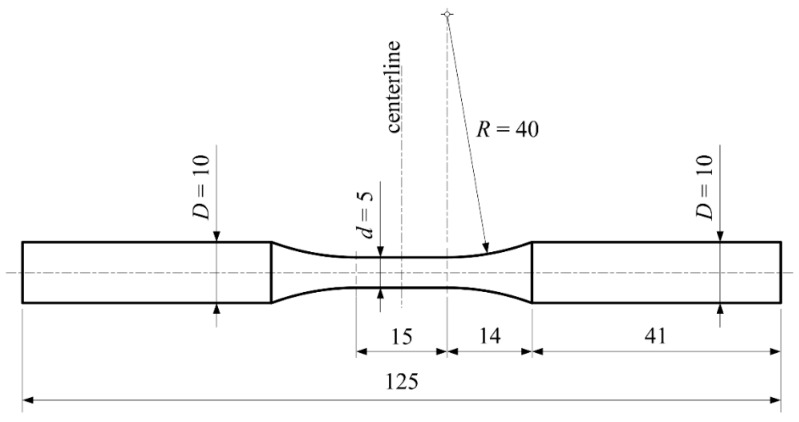
Specimen’s geometry (mm): fatigue test.

Finally, specimens used in fatigue tests, Figure 3, were machined in accordance with the ASTM: E 466-15 standard. All mentioned standards can also be found in the annual book [12].

## 3. Experimental Results and Discussion

Results of this experimental research related to the mechanical behavior of material X46Cr13 are presented in the form of diagrams and/or tables. These experimental results include mechanical properties, creep behavior, impact energy and the fatigue of the considered material. 

### 3.1. Uniaxial Tensile Tests

#### Engineering Stress-Strain Diagrams and Mechanical Properties versus Temperature

**Figure 4:**
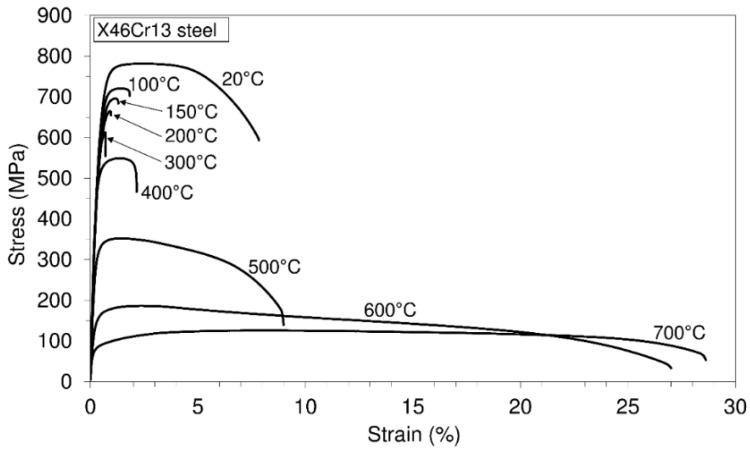
Engineering stress-strain diagrams at different temperatures: X46Cr13 steel.

For the results of uniaxial testing of the considered material at different temperatures, engineering stress-strain diagrams were obtained, as in Figure 4. It is worthwhile to point out that several tests were made at each testing temperature, but the resulting diagrams did not differ much; thus, Figure 1 shows the results of the first test for each of the tested temperatures. Furthermore, in the same manner, Table 2 evaluates the numerical values of the mechanical properties related to the first test at each testing temperature.

**Figure 5:**
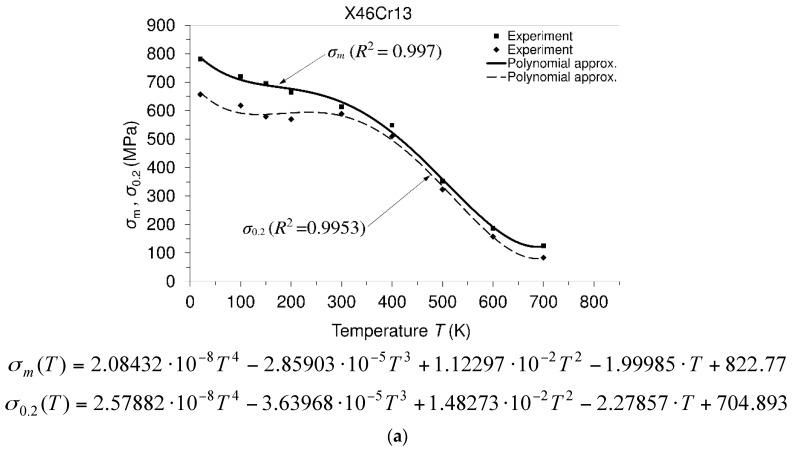

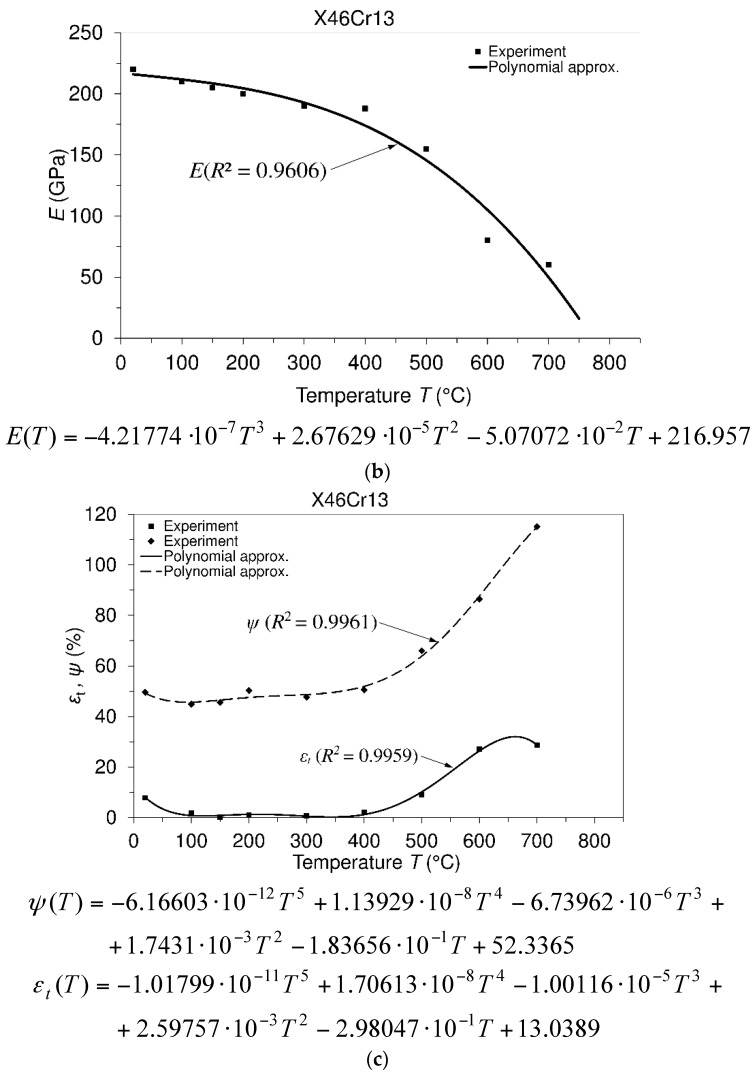
Material properties versus temperature: X46Cr13 steel. (**a**) Mechanical properties ($\sigma_{m}$, ultimate tensile strength; $\sigma_{0.2}$, 0.2 offset yield strength) versus temperature; (**b**) modulus of elasticity (E) versus temperature; (**c**) total elongation ($\varepsilon_{t}$) and reduction in area (ψ) versus temperature.

Figure 5 presents the temperature dependencies of the mechanical properties. Experimental results are shown by using special characters (▪, ♦), while the continuous dependencies of the considered properties are shown by approximation curves (solid line or dashed line). Since these approximations describe the temperature dependence of experimentally-obtained results with some degree of accuracy, the so-called coefficient of determination *R*2 is introduced. It is a measure of accordance between experimental results and mentioned polynomial approximation, and it serves as the statistics that gives the necessary information about how fit a model is [13].

Based on the experimental results, it is visible that the considered steel possesses high ultimate tensile strength (781.7 MPa) and high yield strength (657.5 MPa) at the room testing temperature. Furthermore, this steel exhibits a high level of the modulus of elasticity (220 GPa/20 °C).

Furthermore, with an increase in temperature, all of the mentioned properties decrease. However, even at a temperature of 500 °C, the ultimate tensile strength (352 MPa) and yield strength (323 MPa) may be treated as properties of a high level. In addition, it is visible that after the temperature of 500 °C, the strength decreases quite quickly. On the other hand, the strains that are reduced between the room temperature and the temperature of 500 °C increase after this temperature very quickly. A drop in the strains in the mentioned range of temperatures may be treated as a consequence of dynamic strain aging, which is a hardening phenomenon. When the effect of serrations at the stress-strain curve is not visible, then a strain aging phenomenon can be marked by lower strain rate sensitivity. Dynamic strain aging causes a minimum variation of ductility with temperature changes, and a plateau in strength can be seen. If this material is compared with the 42CrMo4 steel, [14], which can also be used for manufacturing of the shaft, then it can be said as follows. Steel X46Cr13 is martensitic stainless steel (0.442 C; 13.05 Cr), while steel 42CrMo4 (0.45 C; 1.06 Cr) is a low alloy steel, and thus, steel X46Cr13 is very resistant to corrosive environmental conditions. In terms of the strength of the material, steel X46Cr13 (ultimate tensile strength: 781.7 MPa/20 °C; yield strength: 657.5 MPa/20 °C) is much better than 42CrMo4 steel (ultimate tensile strength: 617 MPa/20 °C; yield strength: 415 MPa/20 °C).

### 3.2. Uniaxial Short-Time Creep Tests and Creep Modeling

Generally, testing of the materials to creep can be classified into two groups: short-term and long-term testing. In any case, long-term testing is very expensive. In accordance with the possibilities of available equipment and the cases in which the short-term tests may be of interest, this study considers short-term creep. On the other hand, it is useful to simulate/model material creep behavior for those materials for which the behavior of creep in certain conditions is already known. 

When creep modeling is considered, then the following cases for strain calculation are possible: (a) ε=ε(t);T=const, σ=const; (b) ε=ε(σ,t);T=const (i.e., $T=const:\;\sigma_{1\left(T\right)}=const,\;\sigma_{2\left(T\right)}=const,\ldots ..)$; (c) ε=ε(σ,T,t) (i.e., $T_{1}=const:\;\sigma_{1\left(T_{1}\right)}=const,\;\sigma_{2\left(T_{1}\right)}=const,\;\ldots ..;\;T_{2}=const:\;\sigma_{1\left(T_{2}\right)}=const,\;\sigma_{2\left(T_{2}\right)}=const,\;\ldots .)$.

Modeling can be performed using rheological models [15] or with certain equations. In the case of this investigation, the following equation will be used [16]:

(1) $$\varepsilon \left(t\right)=D^{-T}\sigma^{p}t^{r}$$

In Equation (1), the notations stand for: *T*, temperature; σ, stress; *t*, time; and *D*, *p* and *r* are parameters that need to be determined. Equation (1) is intended to model the first stage of the creep (transient creep) and can be applied for any of the above-mentioned cases (a, b, c).

In the case of this research, creep modelling is performed in such a way that:

(1a) ε(t)=ε(σ,T,t)

Thus, the last of the above-mentioned cases of modeling is performed (time-temperature-stress dependence). In accordance with Equation (1a), Equation (1) provides modelling of any considered (desirable) creep process that belongs to any of the considered stress levels within the considered temperature levels. For the purpose of modeling in the literature, it is known as the Bailey–Norton equation [17,18]:

(1b) $$\varepsilon_{c}=k\sigma^{p}t^{r},\;\to \;\varepsilon_{c}=F\left(\sigma \right)f\left(t\right)$$

The parameters (material constants) in this equation need to be obtained on the basis of the considered creep curve. Equation (1b) can be generalized as the product of two functions, as is shown above. Function f(t) represents any suitable time function, and it was observed that power function $t^{r}$ is in good agreement with the experiments. The exponent “*r*” is in general smaller than 0.5 [17]. In this equation, an instantaneous strain can be involved. If Equations (1) and (1b) are compared, it is visible that Equation (1) can be taken as the rearranged Bailey–Norton equation, and in this case, the factor ($D^{-T})$ can take the meaning of “*k*” in the Bailey–Norton equation. On the other hand, when the temperature range and range of stresses within any of the prescribed temperature levels are considered (above-mentioned case “c”), then factor “D” is obtained on the basis of both ranges. The obtained value of factor “D” in this way (the same is valid for other factors) provides modeling of any creep curve within both of the mentioned ranges (temperature and stress), as is visible from Table 3 and Figure 9a. Furthermore, for creep modeling the following equation can be used [18]:

(1c) $$\varepsilon \left(t\right)=ae^{-A/T}\sigma^{p}t^{r}$$

Since this equation is similar to the previously-used Equation (1), the obtained result using it is shown in Table 3 and Figure 9b. In Equation (1c), a, A, p and r are material parameters.

**Figure 6:**
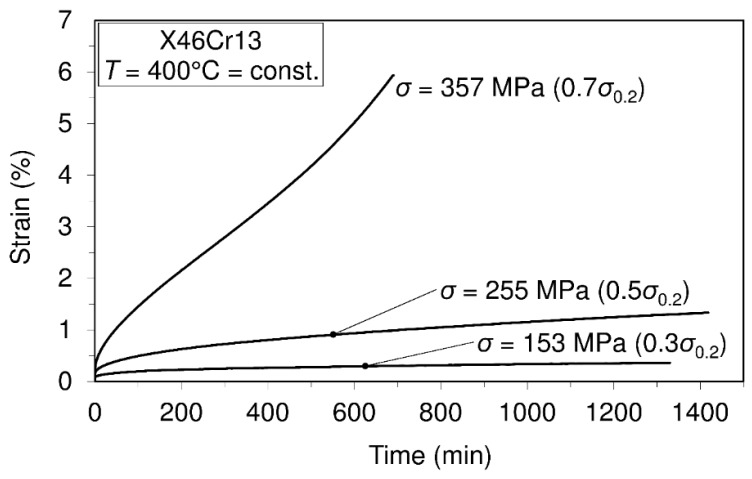
Short-time creep tests of steel X46Cr13 at the temperature of 400 °C.

**Figure 7:**
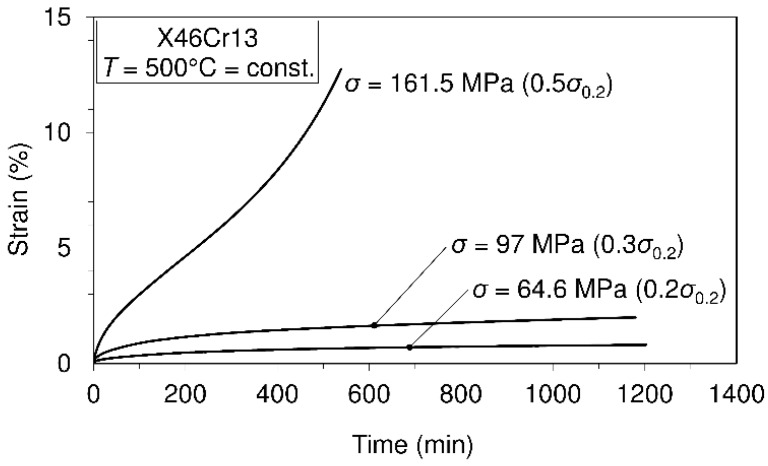
Short-time creep tests of steel X46Cr13 at the temperature of 500 °C.

**Figure 8:**
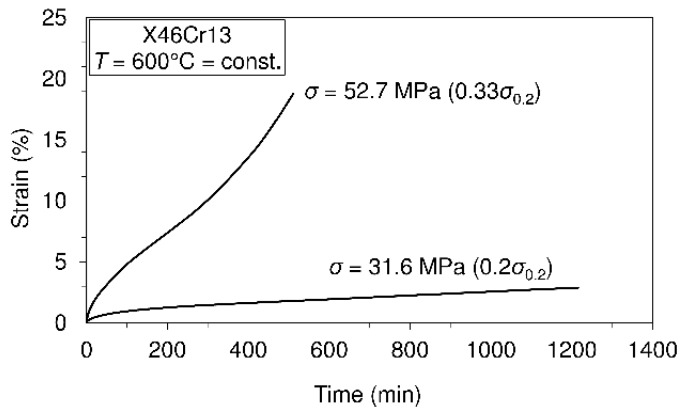
Short-time creep tests of steel X46Cr13 at the temperature of 600 °C.

Creep tests carried out at different temperature levels are presented in Figure 6, Figure 7 and Figure 8.

On the basis of the presented creep curves obtained in short-time creep conditions, some conclusions related to the creep resistance of the tested material may be given. Obviously, this material may be treated as creep resistant at a temperature of 400°C when the applied stress does not exceed 50% of the yield stress at this temperature. Furthermore, it is creep resistant at the temperature of 500 °C when the applied stress does not exceed 30% of the yield stress and, finally, at the temperature of 600 °C when the applied stress does not exceed 20% of the yield stress at this temperature. However, it needs to be said that these test results can give some orientation relating to material creep behavior, but only in the short-time regime of exploitation. If the comparison of this steel with previously-mentioned 42CrMo4 steel [14] is also made with respect to creep resistance, then, in general, it can be said that no significant difference can be seen in the range between 400 °C and 600 °C. However, it can be interesting to note that at the temperature of 600 °C, steel X46Cr13 subjected to stress of 31.6 MPa exhibits the strain of 2.5%, while steel 42CrMo4 subjected to stress of 34 MPa (a little different from 31.4 MPa) exhibits the strain of 5%. This shows that in these conditions, steel 42CrMo4 is less resistant to creep than X46Cr13. In this research, some of the creep curves within the temperature range from 400 °C–600 °C are modeled using the earlier mentioned Equation (1). Selected creep curves are visible in Table 3. This means that using the model presented by Equation (1), any of the creep processes belonging to the mentioned range of temperatures and stress levels can be modeled using the obtained parameters given in Table 3. Of course, using the model presented by Equation (1), any of the mentioned creep curves may also be modeled separately, but in this case, other parameters are valid.

**Figure 9:**
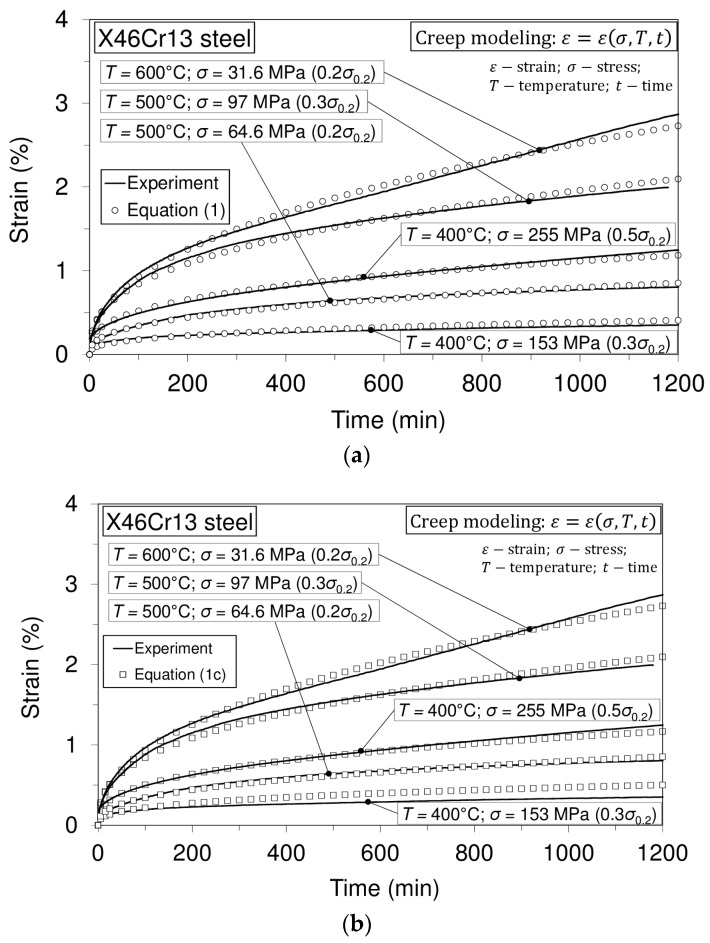
Experimentally-obtained creep curves and modeled creep curves. (**a**) Modeling performed by Equation (1); (**b**) modeling performed by Equation (1c).

In Table 3, creep modeling data are given, while in Figure 9, the creep modeling for selected creep curves is shown.

On the basis of the shapes of the modeled creep curves, it can be said that both models (Equations (1) and (1c)) give quite well-modeled curves. However, based on experience in this kind of modeling, the following should be noted. Since the modeling covers a range of temperatures and a range of stresses, a very large number of (experimental) points of all of the curves is a problem in data processing. Furthermore, the forms of the primary stages of creep curves differ from each other, and the common model is not equally effective for all primary phases of the creep curves. In spite of that, the model provides the simulation of the desired curve, which is an advantage of such a model. It should be pointed out that sometimes, it is difficult to distinguish how far the primary stage of creep extends.

### 3.3. Charpy V-notch Impact Energy and Fracture Toughness Calculation

The service life of the structure depends on operating conditions and material properties. Concerning this, many of the material properties, the design methodology, the maintenance of the structure, etc., can be included in the design procedure. The structure is usually assumed to have been designed and manufactured so that no failure in the material exists. However, many failures can arise in structure service life due to loading, creep, fatigue, etc. In accordance with the design strategy, two of the material properties are usually mentioned, that is yield strength ($\sigma_{0.2}$) and fracture toughness ($K_{Ic}$). Yield strength serves as a criterion for the design of the structure against plastic deformation, while fracture toughness serves as a criterion for the design of structure against fracture. Both of the mentioned properties can be experimentally determined. Since the experimental investigations related to fracture toughness can be expensive and require some efforts in manufacturing specimens, there is a method that is simpler, but that may also serve in the fracture toughness assessment of the considered material. This method consists of deriving the fracture toughness from the fracture impact energy of the material. The impact energy can be determined using the Charpy test method. Fracture toughness can be calculated with the well-known Roberts–Newton equation [19,20,21]:

(2) $$K_{Ic}=8.47\;{\left(\mathrm{CVN}\right)}^{0.63}$$

**Figure 10:**
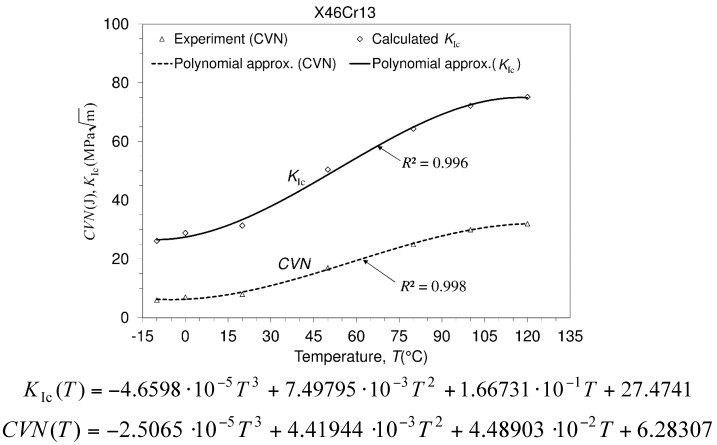
Charpy V-notch impact energy and fracture toughness calculation.

The measured impact Charpy energies are as follows: 6, 6, 6.5/−10 °C; 7, 7, 8/0 °C; 8, 9, 8/20 °C; 17, 17, 17/50 °C; 25, 24, 24.5/80 °C; 30, 29, 29/100 °C; 32, 33, 32/120 5 °C, and they are shown in the Figure 10. Although the Charpy tests are much simpler, the results obtained by the fracture toughness tests are much more reliable. However, fracture toughness testing is also a laboratory method.

### 3.4. Fatigue Tests and Fatigue Limit Calculation

**Figure 11:**
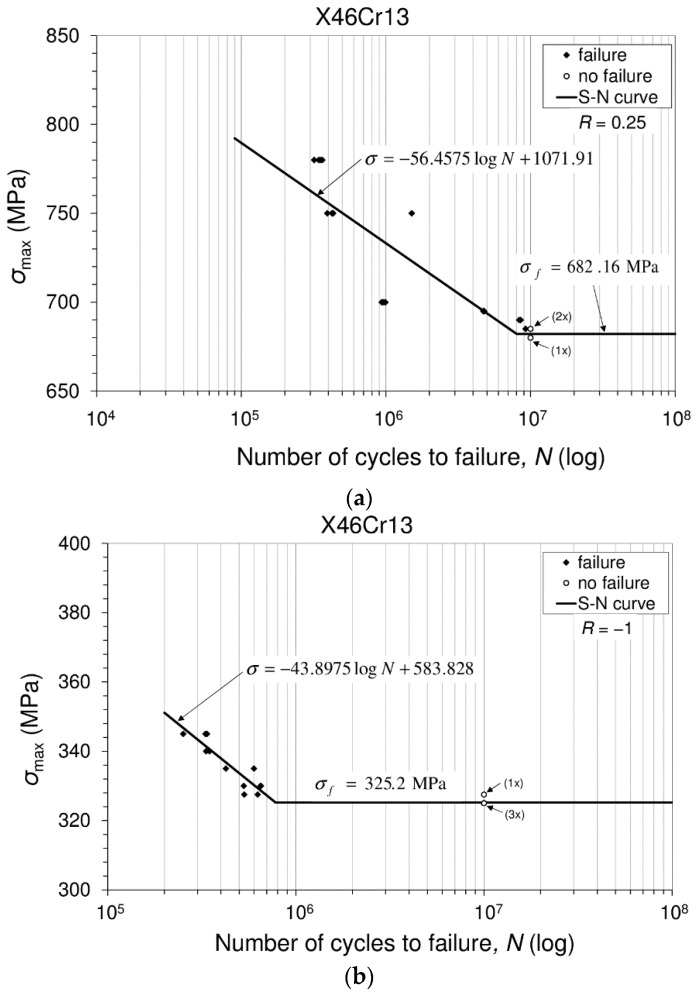
Stress versus number of cycles to failure: experimental data and S–N curve. (**a**) Stress ratio R=0.25; (**b**) stress ratio R=−1.

Fatigue is known as one of the possible mechanical failure modes of engineering structures. Therefore, it is of interest to investigate the resistance of the material to such mechanical failures. Namely, the material subjected to a repeated load (cyclic load) may experience rupture at a stress level significantly lower than the rupture stress corresponding to the same type of monotonic load. Usually, the fracture of an engineering element is defined as the process of damage accumulation due to cyclic loading during its service life [22]. This research is focused on the axial cyclic load. Tests were carried out at room temperature and at two mutually-different stress ratios in accordance with the ISO 12107:2012 (2012) standard [23]. The results of this investigation offer some good information that can be used in structural design against fracture and belong to the so-called stress-life model. Unnotched specimens of the type shown in Figure 3 were subjected to different levels of axial loads for two prescribed stress ratios. For each of the applied stress levels, several tests were carried out since scatter in the number of the cycles to failure may occur. Experimentally-obtained data are placed in the coordinate system; see Figure 11. On the ordinate, data related to the maximum stresses ($\sigma_{max}/\mathrm{MPa}$) are plotted, while on the abscissa, data related to the number of the cycles (N) to failure are plotted. In this way, so-called S–N diagram (stress versus the number of cycles to failure), also known as the Wohler curve, or the fatigue life diagram, can be created, and in this case, this is presented in Figure 11. These diagrams are drawn separately for two stress ratios. Each diagram represents the fatigue behavior of the considered material for one of the prescribed stress ratios. In addition, each diagram consists of the two areas: the one that belongs to the finite fatigue life (region) and the second one that belongs to the infinite fatigue life. Since both parts of the diagrams are presented in a linear form, the diagram consists of one inclined line and one horizontal line. This horizontal line represents the fatigue limit (endurance limit). In engineering practice, for steel alloys, the number of cycles to failure at which the specimen remains unfailed (unbroken) is usually adopted as $10^{7}$ cycles. The fatigue limit (fatigue strength in the infinite fatigue life region, endurance limit), presented in Figure 11 as a horizontal line, can be calculated using a modified staircase method. In both cases, i.e., in the case of stress ratio R=−1, as well as in the case of stress ratio R=0.25, testing was carried out under a decreasing stress regime. Data related to the specimens that have failed and those that have not failed are given in Table 4, and they are needed for the analysis in the staircase method. An analysis of the staircase data for the derivation of the constants is presented in Table 5. The calculation of the constants in accordance with the ISO standard is presented in Table 6.

Data in S–N diagram (curve) related to the failed specimens (♦) and those that did not fail (○) are placed at the appropriate locations according to the number of cycles. Tests were conducted in such a way that the specimen has failed or the test has passed 10 million cycles. However, the S–N diagram in Figure 11 consists of two regions, namely the finite fatigue region represented by the inclined line and the infinite fatigue region represented by the horizontal line.

#### Fatigue Limit Calculation

Fatigue strength in the infinite fatigue life region, known as the fatigue limit (endurance limit), can be calculated in accordance with the modified staircase method. This quantity is drawn as the horizontal line in Figure 11. As was said, analysis in the modified staircase method is also completed on the basis of a decreasing stresses regime. On the basis of the S–N data, the modified staircase method analysis is completed for a particular stress ratio. From the S–N diagram (also Table 5), stress levels used in the modified staircase method are visible. It is also visible that when the staircase modified method for determining the fatigue limit (i.e., in the infinite fatigue regime) is used, the highest level of the stress is $\sigma_{2}$, and this coincides with the lowest stress measured in the finite fatigue regime. In general, the “$\sigma_{i}$” (MPa) stress level corresponds to the “*i*-th” level of testing and to the failure events that occurred, designated as “$f_{i}$”. The step of the stress applied at stress ratio (in the modified staircase method) *R* = 0.25 was d = 5 MPa, and d = 2.5 MPa in the case of R=−1. Data related to the failed and non-failed specimens used in the modified staircase method are shown in Table 4. 

These data are analyzed in a further procedure (Table 5), and constants *A*, *B*, *C* and *D* are determined (Table 6).

The fatigue limit (endurance limit) is defined in accordance with the ISO standard as follows:

(3) $$\sigma_{f\left(P,1-\alpha \right)}={\bar{\mu }}_{y}-k_{\left(P,1-\alpha ,dof\right)}\cdot {\bar{\sigma }}_{y}$$

In Equation (3), the following notations are used:

- ${\bar{\mu }}_{y}$, the mean fatigue strength, which is defined as:
  (4) $${\bar{\mu }}_{y}=\sigma_{0}+d\left(\frac{A}{C}-\frac{1}{2}\right)$$
  where “*d*” is the stress step (i.e., the difference between the neighboring stress levels) (Table 5).
- $k_{\left(P,1-\alpha ,\nu \right)}$, the coefficient for the one-sided tolerance limit for a normal distribution
- ${\bar{\sigma }}_{y}$, the estimated standard deviation of the fatigue strength, which is calculated as:
  (5) $${\bar{\sigma }}_{y}=1.62\cdot d\left(D+0.029\right).$$

In accordance with the standard recommendation of ν =n−1=6, where *n* is the number of items in a considered group. Further, for a desired probability of P=10% and a confidence level (1−α) = 90%, in accordance with Table B1 [23], it is: $k_{\left(P,1-\alpha ,\nu \right)}$ = $k_{\left(0.1;0.9;6\right)}=$ 2.333. In accordance with Equation (4), it is:

$$R=0.25\to {\bar{\mu }}_{y}=\sigma_{0}+d\left(\frac{A}{C}-\frac{1}{2}\right)=680+5\;\left(7/4-1/2\right)=686.25\;\mathrm{MPa}$$

$$R=-1\to {\bar{\mu }}_{y}=\sigma_{0}+d\left(\frac{A}{C}-\frac{1}{2}\right)=325+2.5\;\left(8/5-1/2\right)=327.75\;\mathrm{MPa}$$

or this can be obtained as (Table 4):

$$R=0.25\to {\bar{\mu }}_{y}=\left(680+685+690+685+690+685+690\right)/7=686.4\;\mathrm{MPa},$$

$$R=-1\to {\bar{\mu }}_{y}=\left(325+327.5+330+327.5+330+327.5+330\right)/7=328.2\;\mathrm{MPa},$$

whose amount is similar to the previously-obtained ones.

In accordance with Equation (5), it is:

$$R=0.25\to {\bar{\sigma }}_{y}=1.62\cdot d\left(D+0.029\right)=1.625\cdot 5\;\left(0.1875+0.029\right)=1.75\;\mathrm{MPa}$$

$$R=-1\to {\bar{\sigma }}_{y}=1.62\cdot d\left(D+0.029\right)=1.62\cdot 2.5\;(0.24+0.029)=1.09\;\mathrm{MPa}$$

Finally, the fatigue limit is (3):

$$R=0.25\to \sigma_{f\left(0.1;0.9;6\right)}={\bar{\mu }}_{y}-k_{\left(P,1-\alpha ,\nu \right)}\cdot {\bar{\sigma }}_{y}=686.25-2.333\times 1.75=682.16\;\mathrm{MPa}$$

$$R=-1\to \sigma_{f\left(0.1;0.9;6\right)}={\bar{\mu }}_{y}-k_{\left(P,1-\alpha ,\nu \right)}\cdot {\bar{\sigma }}_{y}=327.75-2.333\times 1.09=325.2\;\mathrm{MPa}$$

**Figure 12:**
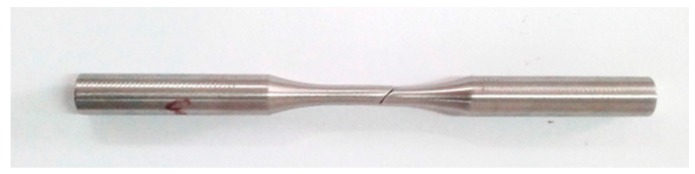
Fractured specimen (from the fatigue process, *R* = 0.25).

One of the fractured specimens is presented in Figure 12.

All fractured specimens at stress ratio R=0.25 and stress ratio R=−1 show the same kind of fracture. The results obtained by means of the fatigue tests give an insight regarding the level of the fatigue limit in comparison with the ultimate strength of the material obtained in static conditions.

### 3.5. Microstructure Analysis

**Figure 13:**
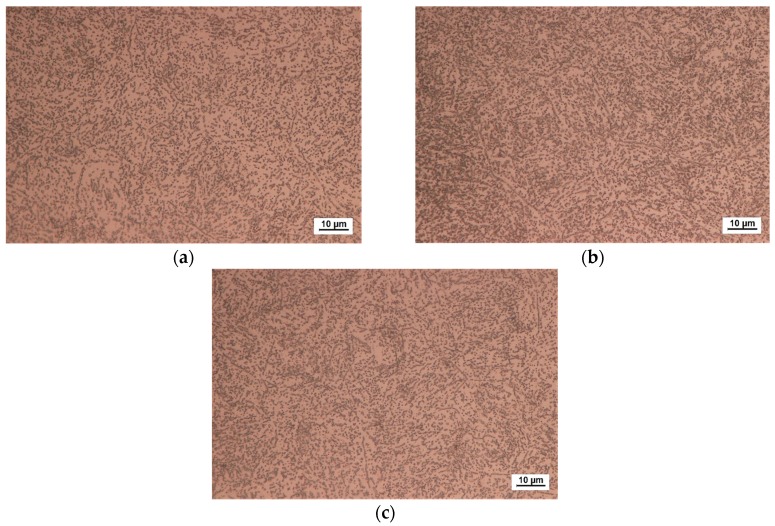
Optical micrograph: steel X46Cr13 (cross-section of the specimen), Aqua regia, 1000×. (**a**) As-received material; (**b**) after the creep process performed at 400 °C, 153 MPa, 1200 min; (**c**) after the creep process performed at 500 °C, 97 MPa, 1200 min.

**Figure 14:**
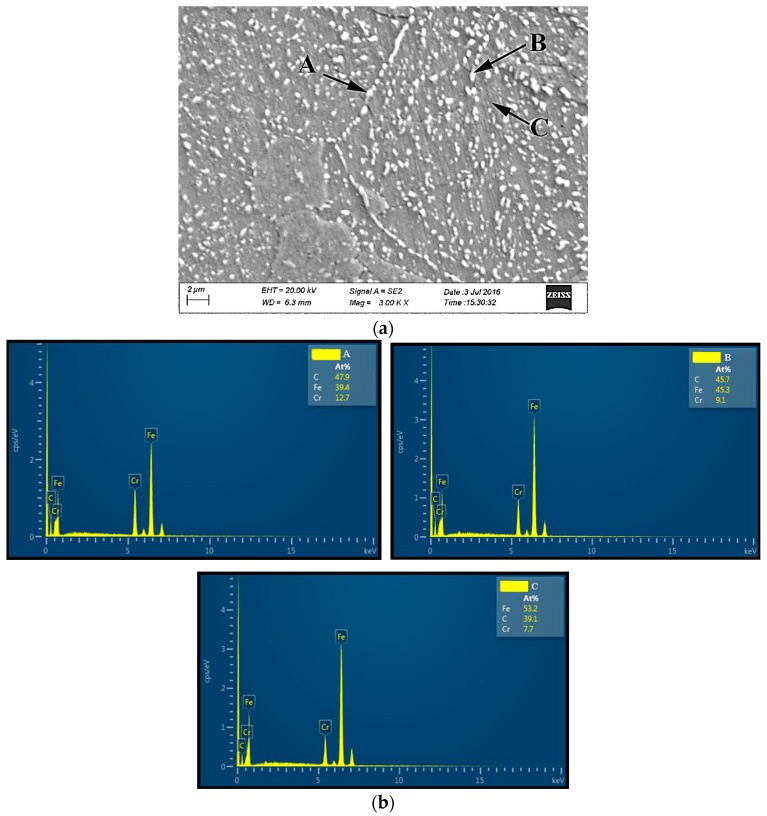
SEM and EDS analysis: X46Cr13 steel under uniaxial fatigue, 776 MPa/194 MPa, stress ratio *R* = 0.25, (average value of cycles to failure: 330,000). (**a**) Fractured surface of the specimen; (**b**) EDS analysis of Points A, B and C.

The basic microstructure analysis of the investigated material was performed on several specimens. An optical microscope was used to investigate the microstructure of as-received material and the microstructure of the material that had previously been exposed to creep. Furthermore, SEM (scanning electron microscopy) was used for the investigation of the microstructure of the material under fatigue. An optical micrograph of the as-received material and optical micrographs of the material that had previously been exposed to creep are presented in Figure 13. Finally, the SEM micrograph of the material exposed to uniaxial fatigue is presented in Figure 14.

Based on the micrograph of the as-received material (Figure 13a), it is visible that the basic microstructure (main phase) is ferrite with spherical pearlites uniformly dispersed. Regarding the material subjected to creep (Figure 13b,c), no visible changes were made. With an electron scanning microscope, the images of a sample (fracture surface of a sample/specimen) can be produced by scanning it with a focused beam of electrons interacting with the atoms in the sample (sample fractured surface). Signals contain information of the sample’s surface topography and composition. EDS (energy dispersive spectroscopy) on the SEM allows for identifying particular elements and their relative proportions (atomic %, for example). In this case, from the insight gained by EDS analysis of Points A, B and C, a synthesis may be deduced that the white phases in the grain boundary and the white spherical particles inside the grain are composed of Fe-Cr-C, which is carbide. They just differ in their contents.

## 4. Conclusions

These investigations were focused on observing the mechanical behavior of the steel 1.4034. In this respect, the mechanical properties of the material in the temperature range of (20 °C–700 °C) are determined, the material creep behavior in the temperature range of (400 °C–600 °C) examined, as well as the fatigue at room temperature for stress ratios R=0.25 and R=−1. The obtained data related to the mechanical properties at room temperature and the highest test temperature of 700 °C are as follows: ultimate tensile strength (781.57 MPa/20 °C; 125.7 MPa/700 °C), yield strength (657.5 MPa/20 °C; 83 MPa/700 °C) and modulus of elasticity (220 GPa/20 °C; 60 GPa/700 °C). These data may serve as good indicators for possible applications of the material for appropriate environmental conditions. Comparing the obtained data related to the mechanical properties of this material with those of steel 42CrMo4, which is also usually used in the manufacturing of shafts, as shown in the text, it is possible to say that this material has better mechanical properties and, besides, is very corrosion resistant. In addition, this material shows a very low total elongation in the temperature domain of 100–400 °C. The obtained data related to the Charpy impact energy (8 J/20 °C; 127 J/200 °C) may also serve for the assessment of fracture toughness. In terms of creep behavior, several creep tests were carried out at different temperatures (400 °C, 500 °C and 600 °C) and at different stress levels within the mentioned temperatures. Some of the results, shown in the form of creep curves, are as follows: (σ= 357 MPa, ε= 6%, t= 700 min/T=400 °C; σ=52 MPa, ε= 20%, t=500 min/T=600 °C). Fatigue testing carried out at different stress ratios and the calculated fatigue limit give an insight into the possible use of the material in the cases of fatigue loadings ($R=0.25:\;\sigma_{f}=682.16\;\mathrm{MPa};R=-1:\;\sigma_{f}=325.2\;\mathrm{MPa})$.

## Figures and Tables

**Table 1:** Material chemical composition: X46Cr13 steel.

Material: X46Cr13 Steel (Chromium Martensitic Stainless Steel)							
Designation							
Steel name				Steel number (Mat. No; W. Nr; Mat. Code)			
EN 10088-3-2005/DIN 17440: X46Cr13; AISI 420; BS: 420S45.				1.4034			
Chemical composition of considered material Mass (%)							
C	Si	Mn	P	S	Cr	V	Rest
0.442	0.375	0.381	0.0121	0.0192	13.05	0.201	85.2997
Mo	Cu	Al	Ti	Pb	Sn	Co	
0.0493	0.108	0.0026	0.0044	0.0004	0.0243	0.031	

**Table 2:** Material properties versus temperature and reduction factors: X46Cr13 steel. ($\sigma_{m}$, ultimate tensile strength; $\sigma_{0.2}$, 0.2 offset yield strength; E, modulus of elasticity).

Temperature T (°C)	Material Properties			Reduction Factors		
	$\sigma_{m}$ (MPa)	$\sigma_{0.2}$ (MPa)	E (GPa)	$k_{1}=\sigma_{m}\left(T\right)/\sigma_{m}\left(20\right)$	$k_{2}=\sigma_{0.2}\left(T\right)/\sigma_{0.2}\left(20\right)$	$k_{3}=E\left(T\right)/E\left(20\right)$
20	781.7	657.5	220	1	1	1
100	720.6	618.4	210	0.922	0.940	0.955
150	695.7	578.5	205	0.889	0.879	0.932
200	664.8	570.1	200	0.850	0.867	0.909
300	613	588.8	190	0.784	0.896	0.864
400	549	510	188	0.702	0.776	0.855
500	352.3	323.3	155	0.451	0.492	0.705
600	186.5	158	80	0,239	0.240	0.364
700	125.7	83	60	0.161	0.126	0.273

**Table 3:** Creep modeling data.

Material	X46Cr13 (1.4034)				
Creep strain-time dependence models: $\varepsilon \left(t\right)=D^{-T}\sigma^{p}t^{r}$ (Equation (1)); and $\varepsilon \left(t\right)=ae^{-A/T}\sigma^{p}t^{r}$ (Equation (1c)) where ε(t)=ε(σ,T,t) Time (min) = 1200, for all creep processes Creep processes were carried out at the temperatures and stresses listed below					
Constant temperature (T °C)	400		500		600
Constant stress level σ(MPa)	153	255	64	97	31
$\sigma =x\cdot \sigma_{0.2}$	x=0.3	x=0.5	x=0.2	x=0.3	x=0.2
Parameters	Parameters (D, p, r) and (a, A, p, r) valid for:				
	*x* = 0.1–0.6		*x* = 0.1–0.4		*x* = 0.1–0.3
D, p, r In accordance with Equation (1)	$D\left(T\right)=1.4300566\cdot 10^{-5}T^{2}-1.3106973\cdot 10^{-2}T+4.0672118$				
	$p\left(T\right)=4.0013503\cdot 10^{-4}T^{2}-3.5889003\cdot 10^{-1}T+81.625941$				
	$r\left(T\right)=1.5594627\cdot 10^{-6}T^{2}-1.0455052\cdot 10^{-3}T+4.9960179\cdot 10^{-1}$				
a, A, p, r In accordance with Equation (1c)	$a\left(T\right)=-9.3283076\cdot 10^{-3}T^{2}+9.3283076T-2.2387938\cdot 10^{3}$				
	$A\left(T\right)=-2.3021939\cdot T^{2}+2.2964057\cdot 10^{3}T-5.4910214\cdot 10^{5}$				
	$p\left(T\right)=-1.024534\cdot 10^{-4}T^{2}+9.7985856\cdot 10^{-2}T-21.166019$				
	$r\left(T\right)=1.7800633\cdot 10^{-6}T^{2}-1.2881659\cdot 10^{-3}T+5.6578198\cdot 10^{-1}$				

**Table 4:** Data for the modified staircase method related to failed (♦) and non-failed (○) specimens obtained from the fatigue tests.

***R* = 0.25**							
**Stress/max** $\sigma_{i}$ **MPa**	**Number of Specimens**						
	**1**	**2**	**3**	**4**	**5**	**6**	**7**
690			♦		♦		♦
685		♦		○		○	
680	○						
***R* = ** −1							
**Stress/max** $\sigma_{i}$ **MPa**	**Number of Specimens**						
	**1**	**2**	**3**	**4**	**5**	**6**	**7**
330			♦		♦		♦
327.5		♦		○		♦	
325	○						

**Table 5:** Data analysis for the modified staircase method (*f* = failed).

***R* = 0.25**				
**Stress/max** $\sigma_{i}$ **(MPa)**	**Number of the level of stress *i***	$f_{i}$	$if_{i}$	$i^{2}f_{i}$
690	2	3	6	12
685	1	1	1	1
680	0	0	0	0
$\sum f_{i,\;}if_{i},i^{2}f_{i}$		4	7	13
***R* = **−1				
**Stress/max** $\sigma_{i}$ **(MPa)**	**Number of the level of stress *i***	$f_{i}$	$if_{i}$	$i^{2}f_{i}$
330	2	3	6	12
327.5	1	2	2	2
325	0	0	0	0
$\sum f_{i,\;}if_{i},i^{2}f_{i}$		5	8	14

**Table 6:** *A*, *B*, *C* and *D* constants calculated in accordance with the ISO standard.

***R* = 0.25**	
**Equation**	**Material: X46Cr13 (1.4034)**
$A=\sum i\cdot f_{i}$	7
$B=\sum i^{2}\cdot f_{i}$	13
$C=\sum f_{i}$	4
$D=\frac{B\cdot C-A^{2}}{C^{2}}$	0.1875
***R* =** −1	
**Equation**	**Material: X46Cr13 (1.4034)**
$A=\sum i\cdot f_{i}$	8
$B=\sum i^{2}\cdot f_{i}$	14
$C=\sum f_{i}$	5
$D=\frac{B\cdot C-A^{2}}{C^{2}}$	0.24
